# Autonomic Conditions in Tinnitus and Implications for Korean Medicine

**DOI:** 10.1155/2013/402585

**Published:** 2013-08-19

**Authors:** Eun Ji Choi, Younghee Yun, Seungyeon Yoo, Kyu Seok Kim, Jeong-Su Park, Inhwa Choi

**Affiliations:** ^1^Department of Otorhinolaryngology of Korean Medicine, Kyung Hee University Hospital at Gangdong, Seoul, Republic of Korea; ^2^Department of Ophthalmology, Otorhinolaryngology and Dermatology of Korean Medicine, College of Korean Medicine, Kyung Hee University, Seoul, Republic of Korea; ^3^Department of Human Informatics of Korean Medicine, Interdisciplinary Programs, Kyung Hee University, Seoul, Republic of Korea; ^4^Kyung Hee Center for Clinical Research and Drug Development, Kyung Hee University, Seoul, Republic of Korea; ^5^Department of Preventive Medicine, Korean Medical College, Kyung Hee University Graduate School, Seoul, Republic of Korea

## Abstract

Tinnitus patients suffer from not only auditory sensations but also physical, mental, and social difficulties. Even though tinnitus is believed to be associated with the autonomic nervous system, changes in autonomic conditions in tinnitus patients are not receiving much research attention. The aims of this study were to investigate the autonomic condition of tinnitus patients and to consider Korean medicine in the treatment of tinnitus with an evidence-based approach. We performed a retrospective chart review and compared the heart rate variability (HRV) parameters of 40 tinnitus patients (19 acute and 21 chronic) and 40 healthy controls. In tinnitus patients, the power of the high frequency component and total power of the HRV significantly decreased (*P* < 0.05), and the low frequency to high frequency ratio significantly increased (*P* < 0.05). There was no significant difference between the acute and chronic patients. When comparing each group with the controls, there was a tendency that the longer the duration of tinnitus was, the larger the observed HRV change was. In conclusion, tinnitus patients have vagal withdrawal and sympathetic overactivity, and chronic tinnitus more strongly affects autonomic conditions than acute tinnitus. This study provides evidence for Korean medical treatments of tinnitus, such as acupuncture and Qi-training, that cause modulation of cardiac autonomic function.

## 1. Introduction

Tinnitus is an auditory phantom sensation experienced when no external sound is present [1]. Its prevalence in Korea has been reported to be 10.5% of the non-noise-exposed population and increases with age [2, 3]. Tinnitus can be categorized according to the duration of the disease. Generally, acute tinnitus is defined as a condition lasting shorter than three months, and chronic tinnitus is considered as a condition lasting longer than three months [4]. Tinnitus is also classified as subjective or objective. In most cases, tinnitus is described as subjective, which cannot be heard by anyone other than the patient [5]. Therefore, subjective tinnitus was the focus of this study.

Patients who experience tinnitus often report associated symptoms, such as emotional difficulties, sleep deprivation, and interference with social interactions [6]. Additionally, the annoyance of tinnitus is not correlated with the acoustic characteristics, but there is a significant correlation with psychological symptoms [7]. Although there are some management options for tinnitus and associated difficulties [8], little high-level evidence exists regarding the efficacy and specificity of the various options currently in use. Therefore, the choice of treatment is now largely in the hands of the individual clinician [9].

The autonomic nervous system (ANS) is a major factor in the difference between simply perceiving tinnitus and being distressed by it [10]. Heart rate variability (HRV) is a noninvasive quantitative and qualitative tool that can be used to evaluate the function of the ANS. HRV studies should enhance our understanding of the physiological phenomena and disease mechanisms of tinnitus. Also, these types of studies may be useful for selecting the most effective therapeutic interventions [11].

There are prior studies regarding tinnitus and the autonomic nervous system. One study reported that heart rate variability (HRV) is suppressed and the sympathetic activity of the ANS is predominant in tinnitus patients [12]. In another study regarding tinnitus severity and neural activity, tinnitus distress correlated positively with sympathetic markers and negatively with parasympathetic markers [13].

However, in previous studies, the number of patients enrolled was small, and the data from control patients did not compensate for age or sex. Also, few studies have evaluated the acute and chronic effects of tinnitus on the ANS. The aims of the present study were to investigate the clinical features of the ANS in tinnitus patients and to define the relationship between the duration of tinnitus and variations in the ANS.

## 2. Materials and Methods

### 2.1. Subjects

#### 2.1.1. Tinnitus Patients

We performed a retrospective chart review of patients with tinnitus who presented at the Department of Otorhinolaryngology of Korean Medicine, Kyung Hee University Hospital in Gangdong, Seoul, Republic of Korea, from January 2008 to February 2012. 

The eligibility criterion for inclusion was first-time Korean adult outpatients presenting for treatment of subjective tinnitus (both bilateral and unilateral). Exclusion criteria were (1) the presence of disease known to affect HRV (cardiovascular, endocrinologic, autoimmune, neurologic, and psychiatric disorders, including alcoholism and polytoxemia), (2) patients with a history of smoking, (3) anticholinergic, antidepressant, or contraceptive pill use for at least four weeks prior to the study, (4) hormone replacement therapy, and (5) pregnancy.

#### 2.1.2. Healthy Controls

The control group was comprised of healthy people who presented for a health checkup at the Health Promotion Center, Kyung Hee University Hospital in Gangdong, Seoul, Republic of Korea, from March 2011 to December 2011.

 Inclusion criteria were (1) Korean adults who underwent an overall health checkup and had a measured HRV and (2) whose age and sex were matched with those of the tinnitus patients. The exclusion criteria were the same as for the tinnitus patients. After screening the healthy controls, we extracted random samples equally matched to the age and sex distributions of the patient group. 

### 2.2. HRV Analysis

Subjects removed any metal attachments from their bodies and were seated on comfortable chairs in a quiet room and then asked to relax for 15 minutes. After the relaxation period, electrocardiography recording was performed for 5 minutes and was then assessed by the SA-3000P (Medicore Inc., Seoul, Republic of Korea). 

The HRV spectrum contains three major components: a very low frequency (VLF, below 0.04 Hz), a low frequency (LF, 0.04–0.15 Hz), and a high frequency (HF, 0.15–0.4 Hz). Recommendations for interpretation of HRV parameters are summarized in Table 1. The HRV was assessed on the basis of the frequency domain measures performed with the use of fast Fourier transformation [11, 14].

### 2.3. Statistical Analysis

SPSS 15.0 for Windows (SPSS Inc., Chicago, IL, USA) was used for data management and statistical analysis. All continuous variables are expressed as mean ± standard deviation (SD). Sex distribution was analyzed using the Chi-square test, and age was evaluated by analysis of variance (ANOVA). HRV variables that were not normally distributed were log-transformed prior to statistical analysis for parametric statistical analysis of nonnormally distributed data by Duan's smearing equation [15]. Transformed HRV variables were analyzed using an independent sample *t*-test or one-way ANOVA between groups. If there were statistically significant differences between groups in ANOVA, the post hoc test was used to identify them. A *P* value less than 0.05 was considered statistically significant.

## 3. Results

### 3.1. Subjects

In the tinnitus patient group, 79 patients were screened, and, of these, 40 (19 acute and 21 chronic) were included in the study. The mean ages of the acute and chronic patients were 45.21 ± 12.46 (SD) years and 47.81 ± 15.87 (SD) years, respectively.

In the healthy control group, we screened 2,114 people, and 40 of these, aged 46.53 ± 14.23 (SD) years, were finally selected as healthy controls. There were no significant differences in the age or sex distribution of the groups. Clinical characteristics of the study participants are summarized in Table 2.

### 3.2. HRV Parameters for Healthy Controls and Tinnitus Patients

The mean values of the HRV measurements are listed in Table 3. In the tinnitus patients, TP and HF were lower, and the LF/HF was significantly higher than in controls. However, VLF and LF were not significantly different between the groups. 

### 3.3. HRV Parameters for Healthy Controls and Acute and Chronic Tinnitus Patients

The values of the HRV parameters, divided into acute and chronic tinnitus patients, are summarized in Table 4. No significant differences were found between the acute and chronic patients. When compared to controls, only the HF was significantly decreased in acute patients; however, in chronic patients, the HF was significantly, decreased and the LF/HF was significantly increased.

## 4. Discussion

We investigated the autonomic condition in tinnitus patients as compared with healthy controls, as well as the acute and chronic effects of tinnitus on the ANS. Considering the differences in autonomic conditions between tinnitus patients (combined acute and chronic tinnitus patients) and healthy controls, the TP and HF were significantly decreased, and the LF/HF was significantly increased in tinnitus patients. A decreased TP could reflect a depressed state of the overall HRV, which has also been reported in several diseases [11] and stressful conditions [16]. A reduced HF can indicate reduced parasympathetic activity. An increased LF/HF could be due to either increased sympathetic autonomic modulation or decreased parasympathetic activity [17]. In the current series, the LF was nearly unchanged, but the HF was decreased in tinnitus. Therefore, the increase of the LF/HF is mainly due to reduction of parasympathetic activity, and this may cause sympathomimetic symptoms to appear more dominant. This result indicates that overall HRV and parasympathetic functions are significantly depressed and sympathetic activity is relatively predominant in tinnitus patients. This is in agreement with a previous study reporting that overall HRV is suppressed and that the activity of the sympathetic branch of the ANS predominates in tinnitus patient [12]. However, the results cannot be adequately compared to prior research because studies on the relationship between tinnitus and autonomic condition are rare. Therefore, understanding that tinnitus is a form of noise perception, we searched for studies regarding noise and HRV and found interesting reports. Previous studies demonstrated reduced parasympathetic activity when listening to a mechanical sound, as opposed to synthesizer music or bird twitters, which have very different frequency components [18]. Because the sound of tinnitus is often a simple frequency, this may be consistent with the results of the present study. In another study, sympathetic activity was increased under exposure to acute noise [19], similar to the current results.

When tinnitus patients were divided into acute and chronic groups, there were no significant differences in HRV parameters. When comparing each group with the controls, in acute tinnitus patients, only the HF was significantly decreased. However, in chronic tinnitus patients, HF was significantly decreased, and the LF/HF was significantly increased. This can be interpreted to mean that chronic tinnitus has a stronger effect on changes in autonomic conditions compared with acute tinnitus.

In Korean medicine, tinnitus patients are treated with various modalities, such as acupuncture, herbal medicine, counseling, and Qi-training [20–22]. Previous studies have revealed that acupuncture at the Sishencong points and Qi-training enhanced cardiac vagal and suppressed sympathetic activities in humans [22, 23]. In a recent study, epifascial acupunctural stimulation at CV17 also increased vagal activity, and the presence of a specific acupunctural point that causes the modulation of cardiac autonomic function is suggested [24]. 

There are several limitations of the current study. The current study was controlled for age- and sex-related differences but not variations in symptom severity. Also, the HRV data were not measured at the same time of the day, so a circadian variation of HRV cannot be excluded. 

To our knowledge, this is one of only a few studies regarding the effects of tinnitus on the autonomic condition. The results of the current study should help to elucidate the associated symptoms leading to a progressive deterioration in quality of life for tinnitus patients and aid in selecting proper Korean medical treatment options for alleviating the accompanying symptoms. 

## 5. Conclusions

According to the HRV analysis of tinnitus patients, we conclude that tinnitus patients have significantly altered HRV parameters compared to healthy controls. Tinnitus reduced parasympathetic activity and chronically induced sympathetic overactivity. Chronic tinnitus had a stronger effect on autonomic changes than acute tinnitus. These findings provide evidence supporting Korean medical treatments for tinnitus, including modalities such as acupuncture and Qi-training, which enhance cardiac vagal and suppress sympathetic activities in humans. The underlying mechanisms and potential applications of Korean medicine for tinnitus warrant further investigation. 

## Figures and Tables

**Table 1:** Interpretation of HRV parameters.

HRV parameter	Interpretation
Total power of the HRV spectrum (TP)	Total strength of ANS (overall state of HRV)
Very low frequency component (VLF)	Sympathetic regulation
Low frequency component (LF)	Both sympathetic and parasympathetic activities
High frequency component (HF)	Parasympathetic activity and the frequency of respiration
Ratio of absolute LF to HF power (LF/HF)	Sympathetic-parasympathetic balance

**Table 2:** Sex and age distributions of the study participants.

Variables	Healthy controls (*n* = 40)	Tinnitus patients		*P* value
		Acute (*n* = 19)	Chronic (*n* = 21)	
Sex				
Male	20	11	9	0.637
Female	20	8	12	
Age, y (mean ± SD)	46.53 ± 14.23	45.21 ± 12.46	47.81 ± 15.87	0.848
Age subgroup				
19–29 y	7	3	4	
30–39 y	3	2	1	
40–49 y	10	5	5	
50–59 y	13	7	6	
60–69 y	5	2	3	
>70 y	2	0	2	

Data are *n* or mean ± SD. Sex distribution was evaluated using the Chi-square test. Age was evaluated by analysis of variance (ANOVA). Statistical significance was set at a *P* value < 0.05.

**Table 3:** HRV parameters for healthy controls versus tinnitus patients.

HRV parameter	Healthy controls	Tinnitus patients	*P* value
TP (ms^2^)	7.49 ± 0.99	6.92 ± 1.14	0.020*
VLF (ms^2^)	6.37 ± 0.92	6.09 ± 1.21	0.256
LF (ms^2^)	5.98 ± 1.19	5.54 ± 1.27	0.114
HF (ms^2^)	6.41 ± 1.19	5.13 ± 1.43	0.000**
LF/HF	0.94 ± 0.11	2.48 ± 3.47	0.008**

HRV data were log-transformed prior to statistical analysis. The values given are the mean ± SD. Statistical significance was set at a *P* value < 0.05, using independent sample *t*-test. TP: total power; VLF: very low frequency; LF: low frequency; HF: high frequency; LF/HF: ratio of absolute LF to HF power; *P < 0.05; **P < 0.01.

**Table 4:** HRV parameters for healthy controls and acute and chronic tinnitus patients.

HRV parameter	Healthy controls	Tinnitus patients		*P* value
		Acute	Chronic	
TP (ms^2^)	7.49 ± 0.99	6.99 ± 1.23	6.86 ± 1.09	0.063
VLF (ms^2^)	6.37 ± 0.92	6.18 ± 1.32	6.01 ± 1.12	0.461
LF (ms^2^)	5.98 ± 1.19	5.52 ± 1.36	5.56 ± 1.22	0.288
HF (ms^2^)	6.41 ± 1.19^a^	5.31 ± 1.34^b^	4.97 ± 1.52^b^	0.000**
LF/HF	0.94 ± 0.11^a^	1.85 ± 2.13^a, b^	3.05 ± 4.32^b^	0.007*

HRV data were log-transformed prior to statistical analysis. The values given are the mean ± SD. Statistical significance was set at a *P* value < 0.05, using analysis of variance (ANOVA). If there were statistically significant differences between groups, the post hoc test (Duncan) was used to identify them with “^a, b^”. TP: total power; VLF: very low frequency; LF: low frequency; HF: high frequency; LF/HF: ratio of absolute LF to HF power; *P < 0.05; **P < 0.01.

## References

[1] Eggermont JJ, Roberts LE (2004). The neuroscience of tinnitus. *Trends in Neurosciences*.

[2] Heller AJ (2003). Classification and epidemiology of tinnitus. *Otolaryngologic Clinics of North America*.

[3] Koo JW, Lee WC, Kim H (1999). Prevalence of tinnitus and hearing thresholds of a non-noise-exposed population with and without tinnitus. *Korean Journal of Occupational and Environmental Medicine*.

[4] Davis A, Rafaie AE, Tyler RS (2000). Epidemiology of tinnitus. *Tinnitus Handbook*.

[5] Martinez Devesa P, Waddell A, Perera R, Theodoulou M (2007). Cognitive behavioural therapy for tinnitus. *Cochrane Database of Systematic Reviews*.

[6] Shargorodsky J, Curhan GC, Farwell WR (2010). Prevalence and characteristics of tinnitus among US adults. *American Journal of Medicine*.

[7] Luxon LM (1993). Tinnitus: its causes, diagnosis, and treatment. *British Medical Journal*.

[8] Han BI, Lee HW, Kim TY, Lim JS, Shin KS (2009). Tinnitus: characteristics, causes, mechanisms, and treatments. *Journal of Clinical Neurology*.

[9] Hoare DJ, Hall DA (2011). Clinical guidelines and practice: a commentary on the complexity of tinnitus management. *Evaluation and the Health Professions*.

[10] Jastreboff PJ, Hazell JW (2004). *Tinnitus Retraining Therapy: Implementing the Neurophysiological Model*.

[11] Malik M, Bigger JT, Camm AJ (1996). Heart rate variability: standards of measurement, physiological interpretation, and clinical use. *Circulation*.

[12] Datzov E, Danev S, Haralanov H, Naidenova V, Sachanska T, Savov A (1999). Tinnitus, heart rate variability, and some biochemical indicators. *International Tinnitus Journal*.

[13] van der Loo E, Congedo M, Vanneste S, de Heyning PV, de Ridder D (2011). Insular lateralization in tinnitus distress. *Autonomic Neuroscience*.

[14] Yasuma F, Hayano J-I (2004). Respiratory sinus arrhythmia: why does the heartbeat synchronize with respiratory rhythm?. *Chest*.

[15] Duan N (1983). Smearing estimate: a nonparametric retransformation method. *Journal of the American Statistical Association*.

[16] Matthews S, Jelinek H, Vafaeiafraz S, McLachlan CS (2012). Heart rate stability and decreased parasympathetic heart rate variability in healthy young adults during perceived stress. *International Journal of Cardiology*.

[17] Björ B, Burström L, Karlsson M, Nilsson T, Näslund U, Wiklund U (2007). Acute effects on heart rate variability when exposed to hand transmitted vibration and noise. *International Archives of Occupational and Environmental Health*.

[18] Yanagihashi R, Ohira M, Kimura T, Fujiwara T (1997). Physiological and psychological assessment of sound. *International Journal of Biometeorology*.

[19] Tzaneva L, Danev S, Nikolova R (2001). Investigation of noise exposure effect on heart rate variability parameters. *Central European Journal of Public Health*.

[20] Kim YB (2002). Clinical observations for evaluation protocol on tinnitus. *Korean Journal of Oriental Physiology & Pathology*.

[21] Jeon JH, Choi EH, Kim YI (2008). Acupuncture combined with herbal medicine for the treatment of tinnitus: clinical study in 11 cases. *The Journal of Korean Acupuncture & Moxibustion Society*.

[22] Lee MS, Huh HJ, Kim BG (2002). Effects of Qi-training on heart rate variability. *American Journal of Chinese Medicine*.

[23] Wang JD, Kuo TBJ, Yang CCH (2002). An alternative method to enhance vagal activities and suppress sympathetic activities in humans. *Autonomic Neuroscience*.

[24] Kurono Y, Minagawa M, Ishigami T, Yamada A, Kakamu T, Hayano J (2011). Acupuncture to Danzhong but not to Zhongting increases the cardiac vagal component of heart rate variability. *Autonomic Neuroscience*.

